# 

**Published:** 1881-10

**Authors:** 


**TERMS.—50 Cents a Year. Address the Bistoury, Elmira, N. Y.**

### SUBSCRIPTIONS RECEIVED UP TO OCTOBER 1st, 1881.

NEW YORK—Dr P S Smith, Clinton Smith, Dr F D Parker, Marcus Brown, Dr A P Bush $2, Clara Spaulding, Dr. John Pierce $1.50, Dr A P Clark $3, Dr A L Samuels $1, Dr F Swartz $2, Dr Harris Beers $2, Dr Jas Davis $1, Dr A D Lobb $1.

PENNSYLVANIA—L C Logwell $1.50, W C Packer, J S Smith, Dr C M Martin, Dr D Hobart $4, Dr Miles Millner $3, Dr Frank Haas $1.50, Mrs A Shindel, Dr Thos Lyon, Dr Harvey Mead $2.

ALABAMA—Dr John Comstock $2, Dr L D Hickock $1

DISTRICT COLUMBIA—F J Heiberger $1, T S Hawley $1.

DELAWARE—Dr E J Smyth.

FLORIDA—Dr P Grosbeck $2, Dr B Hines $1.50.

GEORGIA—Dr L M Montague $4, Jas J Gordey, Dr H D Floyd $2, Dr O Loomis $1.

ILLINOIS—Dr Frank Hendy $2, Dr S D Coons $2, Dr Jas Bloomer, Drs Platt & Herrington $2.

INDIANA—Dr H Hosford $1, Dr Geo O'Reilley $1, Dr P H Kline $2.

KENTUCKY—Dr T D Newberry, Dr A DeWitt $1.

MISSOURI—Dr S Wallace, Dr H Fremont, Dr A J McDowell $2, Dr E Hodges $2, Dr. Abel Hunt $2, Dr Geo J Simonds $1.50.

NO NAME—Dr P J Kline $2.

